# Sex Differences in Mortality After CABG Surgery

**DOI:** 10.5935/1678-9741.20150073

**Published:** 2015

**Authors:** José Albuquerque de Figueiredo Neto, Lea Coutinho Barroso, Joana Kátya Veras Rodrigues Sampaio Nunes, Vinicius José da Silva Nina

**Affiliations:** 1 Universidade de São Paulo (USP), São Paulo, SP, Brazil; 2 Universidade Federal do Maranhão (UFMA), São Luís, MA, Brazil

**Keywords:** Myocardial Revascularization, Cardiovascular Surgical Procedures, Coronary Artery Bypass

## Abstract

**INTRODUCTION:**

Numerous studies have shown that women undergoing coronary artery bypass graft surgery present higher mortality rate during hospitalization, and often complications when compared to men.

**OBJECTIVE:**

To compare the mortality of men and women undergoing coronary artery bypass graft surgery and identify factors related to differences occasionally found.

**METHODS:**

Retrospective cohort study conducted with 215 consecutive patients who underwent coronary bypass surgery.

**RESULTS:**

Women had a higher average age. Low body surface and dyslipidemia were more prevalent in women (1.65 *vs*. 1.85, *P* <0.001: 53% *vs*. 30%, *P* =0.001), whereas history of smoking and previous myocardial infarction were more prevalent in men (35% *vs*.14.7%, *P* =0.001; 20% *vs*. 2.7%, *P* =0.007). Regarding complications in the postoperative period, there was a higher rate of blood transfusions in women. The overall mortality rate was 5.6%, however there was no statistically significant difference in mortality between men and women. It was observed that among the patients who died, the average body surface area was lower than that of patients who did not have this complication.

**CONCLUSION:**

There was no difference in mortality between the sexes after coronary artery bypass graft in this service.

**Table:** 

**Abbreviations, acronyms & symbols**	
AMI	= Acute myocardial infarction
ARF	= Acute renal failure
CABG	= Coronary artery bypass graft
CPB	= Cardiopulmonary bypass
CVA	= Cerebrovascular accident
HUPD	= Hospital Universitário Presidente Dutra
SUS	= Unified Health System

## INTRODUCTION

The number of patients suffering from coronary artery disease grows progressively around the world, because of the longer survival after treatment of acute ischemic frames and the largest number of diagnoses made and due to the high prevalence of risk factors and specific situations, such as the greater participation of women in the economy and the largest number of elderly in the general population^[1]^.

Some estimates show that women in their 40s are at risk of developing cardiovascular disease over the 32% order of life, and despite the knowledge that cardiovascular disease as the leading cause of death have increased only about 55% women identify heart disease as their greatest health risk^[2]^. According to Ministry of Health, infarction and cerebrovascular accident (CVA) are the leading causes of death in women over 50 years in Brazil^[3]^.

The coronary artery bypass graft (CABG) has proven effective method for the treatment of coronary artery disease, prolonging the lives of patients^[4]^ in Brazil between the years 2005 and 2007. The Unified Health System (SUS) carried out 63,529 isolated CABGs, 33% of these surgeries performed on women^[5]^.

Numerous studies have shown that women undergoing CABG surgery present during hospitalization, higher mortality rate and often complications when compared to men^[6,7]^. Another factor related to higher female mortality is the lowest use of arterial grafts in women^[8]^.

In other studies after correction for age and risk factors, the female ceases to be an independent factor for increased hospital mortality, making believe that these factors, and not sex itself, those responsible for higher surgical risk and higher mortality^[9,10]^.

Our objectives were: 1) to compare morbidity and hospital mortality of men and women undergoing isolated CABG and 2) to identify preoperative, intraoperative and postoperative factors related to possible differences between the two sexes.

## METHODS

### Type of Study

We developed a retrospective cohort observational study with patients undergoing isolated CABG in Hospital Universitário Presidente Dutra (HUPD).

### Sample

In this study, 312 patients underwent isolated CABG surgery, and 97 were not included because CABG was associated with other procedures and/or submission of incomplete data records. The final sample consisted of 215 patients.

### Variables

#### Preoperative data

Data collection was conducted through analysis of medical records, registered in record protocol. Demographic data were collected: age, sex, weight and height (body surface index calculation), and presence of comorbidities such as: 1) hypertension: background described in history, antihypertensive medication or being admitted to the hospital, leading to a greater systolic or equal to 140 mmHg and/or diastolic pressure or equal to 90 mmHg^[11]^; 2) background described in the anamnesis, oral hypoglycemic medication and/or insulin or the examination of preoperative blood reveal glucose in fasting greater than or equal to 126 mg/dl^[12]^; 3) smoking: current or stopped if there were less than 1 year, 4) chronic obstructive pulmonary disease: when cited in the background; 5) prior neurological symptoms and/or a history of CVA; 6) dyslipidaemia: history reported by the patient or introduce changes in admission exams.

Patients were also assessed for the presence of angina and congestive heart failure.

The extent and severity of coronary heart disease were analyzed by conventional angiography.

#### Intraoperative data

Intraoperative variables were evaluated as extracorporeal circulation time.

#### Postoperative data

Complications were: increased thoracic bleeding, defined as one with an average above 500 ml/^[13]^, reoperation for bleeding, hemodynamic instability, cardiac arrhythmias, prolonged mechanical ventilation, when more than 48 hours, CVA, acute myocardial infarction (AMI), pneumonia, wound infection, mediastinitis, sepsis, congestive heart failure and acute renal failure (ARF), defined as serum creatinine increase of 0.5 mg/dl for patients with lower baseline creatinine 1.3 mg/dl and increased by least 50% for those with creatinine greater than 1.3 mg/dl^[14]^.

#### Ethical aspects

The study was approved by the Ethics Committee of the HUPD (Protocol 006 245/2008-00), according to the National Council of Health 196/96 involving humans.

#### Statistical analysis

Comparisons of qualitative variables were established by the chi-square test, Fisher's exact test if the expected frequencies less than 5, and the quantitative variables were compared using ANOVA, Mann-Whitney in case of no normality. Univariate analysis using logistic regression method was performed to determine the risk factors associated with the occurrence of death in women after surgery. In order to determine the overall association of the variables with the incidence of death, variables with *P* <0.05 in the univariate analysis were tested using multivariate logistic regression. The normality test applied was the Shapiro-Wilker test. We used Stata^®^ version 10.

## RESULTS

In all, 215 patients were analyzed, 75 (35%) were female and 140 (65%) were male. Women had higher average age than men (64.4 *vs*. 62.8), as shown in Table 1.

**Table 1:** Demographic characteristics, risk factors and comorbidities by sex in patients undergoing CABG in HUPD, 2007-2008.

Variables		Gender		Total	*P*-value
		Male	Female		
Age	42 - | 50	11	10	21	0.027
	50 - | 60	45	13	58	
	60 - | 70	56	27	83	
	70 - | 82	28	25	53	
Dyslipidemia	Yes	42	40	82	0.001
	No	98	35	133	
Previous AMI	Yes	28	2	30	0.000
	No	112	73	185	
Peripheral vascular disease	Yes	5	2	7	0.534
	No	135	73	208	
Tobacco	Yes	49	11	60	0.001
	No	91	64	155	
Total		140	75	215	

AMI=acute myocardial infarction; CABG=coronary artery bypass graft; HUPD= Hospital Universitário Presidente Dutra

In Table 2, women had a lower body surface than men (1.65 *vs*. 1.85) and higher prevalence of dyslipidemia, 53% *vs* 30%, *P* =0.001. Smoking was more common in men (35% *vs*. 14.6%, *P* =0.001), as well as occurrence of previous infarction (20% *vs*. 2.6%, *P* <0.001).

**Table 2:** Risk factors and comorbidities by sex in patients undergoing CABG in HUPD, 2007-2008

Variables		Sex		Total	*P*-value
		Male	Female		
Angina	Yes	80	45	125	0.398
	No	60	30	90	
Previous Cerebral Vascular Accident	Yes	9	1	10	0.820
	No	131	74	205	
Angioplasty with stent implantation	Yes	22	3	25	0.120
	No	116	68	184	
	Information ignored	2	4	6	
Commitment coronary	Left main coronary artery lesion	32	19	51	0.219
	Uniarterial	15	6	21	
	Biarterial	21	18	39	
	Triarterial	69	28	97	
	Missing information	3	4	7	
Total		140	75	215	

CABG=coronary artery bypass graft; HUPD=Hospital Universitário Presidente Dutra

The coronary angiographies were analyzed in all patients and 97 (46.6%) patients had multivessel coronary angiography and 51 (24.5%) lesions possessed lesion in the left main coronary artery, and had no relationship between the number of vessels affected.

Regarding intraoperative data, and as can be seen in Table 3, the cardiopulmonary bypass (CPB) time was longer in men (47% of men showed CPB time greater than one hundred minutes *vs*. 38%, *P* =0.033).

**Table 3:** Intraoperative of patients undergoing CABG according to gender variables in HUPD, 2007-2008.

Variables		Sex		Total	*P*-value
		Male	Female		
Prolonged Cardiopulmonary bypass time	Yes	137	72	209	0.349
	No	3	3	6	
Intraoperative transfusion	Yes	79	57	136	0.003
	No	61	18	79	
Internal Mammary use	Yes	133	64	197	0.160
	No	7	11	18	
Revascularization	Full	110	55	165	0.213
	Incomplete	29	17	46	
	Information ignored	1	3	4	
Total		140	75	215	

CABG=Coronary artery bypass graft; HUPD=Hospital Universitário Presidente Dutra

There was no statistically significant difference between men and women in the occurrence of postoperative complications, except for the increased need for blood products transfusion during surgery in women (75% *vs*. 56%, *P* =0.003), as shown in Table 4.

**Table 4:** Postoperative of patients undergoing CABG according to gender variables in HUPD, 2007-2008.

Variables		Sex		Total	*P*-value
		Male	Female		
Postoperative transfusion will	Yes	93	50	143	0.764
	No	46	25	71	
	Do not apply	1	0	1	
Death	Yes	8	4	12	0 588
	No	132	71	203	
Mechanical Ventilation> 48h	Yes	6	2	8	0.660
	No	65	34	99	
	Do not apply	67	39	106	
	Information ignored	2	0	2	
Bleed	Yes	37	8	45	0.190
	No	37	28	65	
	Do not apply	66	39	105	
Hemodynamic instability	Yes	35	19	54	0.690
	No	39	17	56	
	Do not apply	66	39	105	
Arrhythmia	Yes	20	12	32	0.633
	No	54	24	78	
	Do not apply	66	39	105	
CICr1	Have kidney disease	33	21	54	0.141
	Do not have kidney disease	80	33	113	
TOTAL		113	54	167	

CABG=Coronary artery bypass graft; HUPD=Hospital Universitário Presidente Dutra

The overall mortality was 5.58% (12 patients). The overall hospital mortality was 5.7% for men and 5.4% for women, without statistically significant difference.

## DISCUSSION

In our analysis, women represented a significant portion of the sample (about 35% of patients). The average age of women was higher than men, a fact confirmed in other studies^[4,15,16]^. Vaccarino et al.^[4]^, in a comparative analysis of 1,113 patients undergoing CABG, also noted this phenomenon, an explanation for this occurrence is because the onset of symptomatic coronary artery disease in women is delayed by up to 10 to 15 years after menopause, thus women are referred for surgery on an older men^[17]^.

In our study, women had a higher prevalence of dyslipidemia, a fact confirmed by other authors^[10,16]^. Amato et al.^[10]^, in a study conducted between 1999 and 2002, including 2,032 patients also showed higher prevalence of dyslipidemia in women, and the disorder seems to have particular significance for women, worsening the prognosis after CABG during the postoperative period.

The degree of commitment of coronary heart disease was similar in both sexes, except for a few studies that found a higher number of three-vessel disease among men^[8,18,19]^, such as Ennker et al.^[15]^, in analysis between the years 1996 and 2006, in Germany. This profile reproduces exactly further analysis comparing the two sexes^[10,20]^.

Regarding the general population of our study we can conclude that our patients had more severe coronary disease, as 68.8% of patients had triple vessel disease or left main coronary return for injury, which was not observed in other studies^[10,15,16]^, which may be due to lack of access to medical care in our state, the lack of services that perform such surgery and the low socio-cultural level of the population seeking medical care at an advanced stage of coronary disease.

In our study there was no difference as elective or emergency nature of the procedure between the two groups, discordant fact other studies^[15-17]^. Bukkapatnam et al.^[21]^, showed that women are more subjected to emergency procedures, justification for such an event would be a smaller proportion of referral of women, by physicians for diagnostic and therapeutic procedures, so that women who reach surgery do so in an emergency situation and that these may be the reasons for surgical results less satisfactory than men^[1,9]^.

In the analysis of postoperative complication rates, there were no significant differences between men and women, except in the greater rates of transfusion of intraoperative blood products in women (Tables 3 and 4), which was not assessed in other studies, which can be explained by the higher incidence of preoperative anemia in the female population^[22]^ due to own physiological factors of women.

It was also found that the incidence of complications in our environment was high compared to that observed by Amato et al.^[10]^, (51% *vs*. 34%), such that the percentage of patients who developed at least one complication is comparable to that observed in studies whose sample consisted of septuagenarian or older patients^[15-25]^, this fact can be explained by the criteria adopted in this study, however, there was no impact on patient mortality.

Overall hospital mortality in this study was similar to the national average^[5]^ and of the country's Northeast. In our study, there was no significant difference in mortality between men and women, a fact confirmed by Ennker et al.^[15]^, in a study of 12,606 patients undergoing myocardial revascularization after adjusting for preoperative risk factors. In the analysis of Amato et al.^[10]^, the female also not proved to be an independent prognostic factor for death.

However, contrary to the vast majority of studies showing increased female mortality , Bukkapatnam et al.^[21]^ observed after multivariate analysis, despite statistical adjustments, the relative risk of death in CABG after surgery in women is 1.65. The justifications for such an event are: difficulties with surgical technique due to the smaller size of the coronary artery, which would have a greater propensity to thrombosis, especially near the suture line and less use of arterial grafts, this technique protects against graft failure^[21]).^

Although we do not find a higher female mortality in our study, we found that the deaths occurred in patients with a body surface who evolved without this complication (1.65 *vs*. 1.85, *P* <0.001). This finding agrees with the study by Ennker et al.^[15]^, which supports the theory that sex is not an independent risk factor for mortality, but the lower body surface affects the outcome.

## CONCLUSION

In conclusion, there was no difference in mortality between men and women in CABG during the postoperative period in our service. We also note that overall mortality in the postoperative period of CABG was similar to the national average, despite the difficulties faced by our service situated in one of the states with the lowest Human Development Indexes, geographically far from traditional training centers.

**Table:** 

**Authors' roles & responsibilities**	
JAFN	Study design; analysis/interpretation of data; manuscript writing/critical review of its content; final approval of the manuscript
LCB	Study design; implementation of projects/experiments; manuscript writing/critical review of its content; final approval of the manuscript
JKVRSN	Analysis/interpretation of data; statistical analysis; manuscript writing/critical review of its content; final approval of the manuscript
VJSN	Study design, statistical analysis; final approval of the manuscript
